# A Novel Approach to Using Spectral Imaging to Classify Dyes in Colored Fibers

**DOI:** 10.3390/s20164379

**Published:** 2020-08-05

**Authors:** G. M. Atiqur Rahaman, Jussi Parkkinen, Markku Hauta-Kasari

**Affiliations:** 1 Computational Spectral Imaging Lab, School of Computing, University of Eastern Finland, FI-80101 Joensuu, Finland; markku.hautakasari@uef.fi; 2 Computational Color and Spectral Image Analysis Lab, Computer Science and Engineering Discipline, Khulna University, Khulna 9208, Bangladesh; 3 Institute of Photonics, University of Eastern Finland, FI-80101 Joensuu, Finland; jussi.parkkinen@uef.fi

**Keywords:** spectral imaging, classification, logistic regression, cultural heritage, dyes, SVM

## Abstract

In the field of cultural heritage, applied dyes on textiles are studied to explore their great artistic and historic values. Dye analysis is essential and important to plan correct restoration, preservation and display strategy in museums and art galleries. However, most of the existing diagnostic technologies are destructive to the historical objects. In contrast to that, spectral reflectance imaging is potential as a non-destructive and spatially resolved technique. There have been hardly any studies in classification of dyes in textile fibers using spectral imaging. In this study, we show that spectral imaging with machine learning technique is capable in preliminary screening of dyes into the natural or synthetic class. At first, sparse logistic regression algorithm is applied on reflectance data of dyed fibers to determine some discriminating bands. Then support vector machine algorithm (SVM) is applied for classification considering the reflectance of the selected spectral bands. The results show nine selected bands in short wave infrared region (SWIR, 1000–2500 nm) classify dyes with 97.4% accuracy (kappa 0.94). Interestingly, the results show that fairly accurate dye classification can be achieved using the bands at 1480nm, 1640 nm, and 2330 nm. This indicates possibilities to build an inexpensive handheld screening device for field studies.

## 1. Introduction

Natural and synthetic dyes have been used historically in clothes, carpets, blankets, tapestries, shrouds, paintings on fabrics etc. to obtain a wide array of different hues [1,2]. However, as both the dyes and the substrate fibers are naturally deteriorating, it is essential to develop effective restoration, preservation and display strategy. Accurate, fast and precise detection and classification of the dyes in such historical objects is thus of substantial importance in the fields of cultural heritage [3,4] and forensic science [5]. Unfortunately, most conventional technologies used for the dye identification process are destructive; damaging to the fugitive objects. There are a number of works where knowing prior information of dye class could be decisive, and eventually would lead to avoid or minimize the damage to the rare pieces of historical objects.

Natural textile fibers like cotton, wool or silk vary in cross-sectional shape, color, surface contour, chemical structure, length and width [6]. Protein is the main component in wool or silk that is animal fiber. Wool is composed of the fibrous protein α-keratin, and the fiber diameters vary from 11 to 100 microns depending on wool sources [7]. Generally wool contains carbon, hydrogen, oxygen, nitrogen and sulphur in different proportions. Wool is chemically viewed as a polymer made of more than 100 amino acids. The 20 common natural α-amino acids are found in the peptide bonds that keep the amino acids together in wool fiber [8]. Differences in physical and chemical properties are basis to identify textile fibers by various technologies. The common purpose of all technologies is to determine as distinctly as possible a fiber’s morphological, optical and chemical properties [6,7,8,9].

The American Society for Testing and Materials (ASTM) recommends infrared (IR) spectroscopy for fiber identification. The absorption, reflection and transmission of light in a fiber depends on the functional groups (bonds) of the fiber. For example, the molecular vibrations of the nonpolar bonds are strong in Raman spectrum and vibrations of the polar bonds in IR spectrum [6]. Note that the fundamental principles of Raman spectroscopy is a scattering process while IR spectroscopy is a pure absorption phenomenon. The wool fiber reflectivity in terms of the ratio K/S of absorption (K) and scattering (S) coefficients following Kubelka–Munk theory is explained in [10]. In contrast to the synthetic fibers the natural fibers have more intra-sample variation in the absorption spectrum.

A review article on using natural dyes in colorization of textile fibers is in [11]. Although wool fiber has native black, brown or different shades of gray as natural color attributed to the amount of melanin, the color and structural characteristics change during storage, scouring, dyeing, bleaching and other finishing processes [12,13,14]. Guesmi in [13] investigated the effects of dye concentration, dye bath pH, salts addition, dyeing temperature, and dyeing time on color strength (K/S) and light fastness. It was revealed that adding different mordants affect the color strength (K/S) of dyed wools according to the order of uses. The Spectral characterization of wool dyed with natural dye can be consulted with [15].

In [2], a number of fiber samples of historical textiles were tested to study various properties of dyes as a function of generic class. In another study [16], 45 samples of museum textiles were investigated to detect synthetic dyes among which only 14 yarns were identified containing the target dye. Ahmed et al. examined dyes of textiles that are of Ottoman age to develop a plan for conservation treatment [17]. Although, in [18], the objective was to analyze natural dyes as it was supposed to be contained in 30 textile samples stored in Mount Athos, synthetic dyes were identified unexpectedly in some of them. Claro et al. investigated 76 samples from Pre-Columbian textiles to study the properties of natural dyes [19]. Samples of length about half a centimeter were collected from five historical Roman textiles to identify the origin of the natural dyes [20]. Similar type of investigation is conducted in forensic science applications regarding the fiber dyes [21,22].

Existing common diagnostic technologies to perform such investigations are high-performance liquid chromatography (HPLC), HPLC coupled with UV-Vis detection, Raman and surfaced-enhanced Raman scattering spectroscopy, X-ray fluorescence etc. The investigations are performed by analyzing the chemical structures at the molecular level to identify the dyes chemically extracted from the fibers [16,18,19,20,23,24]. The accuracy and sensitivity of these techniques cannot be denied. However, most of the technologies are sampling based, elemental and need complex dye extraction and chemical preparation methods [4,25,26]. Moreover, sample collection, preparation and chemical analysis are often challenging due to the deteriorating nature of the materials in the historical objects.

There exist some non-destructive spectroscopic optical technologies based on (diffuse) reflectance, fluorescence, X-ray fluorescence and Fourier transform infrared (FT-IR) [27,28]. However, these technologies are limited by point analysis where the points are selected by human observers. Also point information is inadequate for the analysis of the objects due to the high spatial heterogeneity. Importantly, the target areas of the objects are often not visually detectable due to the alterations in material status, deterioration effects and interventions [28]. Raman and FT-IR spectroscopy can be used for spatial mapping of colorants, but they have also intrinsic technological restrictions [29]. Due to these limitations of spectroscopic techniques the state-of-the-art spectral reflectance imaging is being popular as an alternative. This technique neither needs to remove any sample nor needs any direct contact to the objects. In addition, the operation is fast, in-situ and spatially resolved, the technology therefore is currently drawing attention to investigate the colorants of various art and historical objects [30,31,32,33].

In spectral imaging, the reflectance measurements are made in a series of narrow and contiguous wavelength bands, and such between-band information can be used to characterize the dyes at pixel level. However, the diffuse reflectance in visible to near infrared (V/NIR, 400–1000 nm) or short-wave infrared (SWIR, 1000–2500 nm) wavelength range is broad, thus consists of broad emissions of different molecules of the object under examination [34,35]. As a result, the detection and discrimination of dyes is challenging as the information encoded in measured reflectance spectra are not molecule specific. Rather, the reflectance represents a mixture of information both from the molecules of dyes and the fibers. Fortunately, computational methods are capable to identify the regions in the wavelength scale characterized by the individual material [29].

We argue that, especially in the fields of cultural heritage, preliminary identification of generic dye class by spectral imaging can have considerable advantages. The advantages include minimizing the destruction of the object, making decisions concerning further analytical tools, or deciding on dye extraction methods [25]. However, to the best of our knowledge to the date no work has been reported in the literatures as such. This paper presents a novel approach to classify the fiber dyes as natural or synthetic class using the spectral reflectance of the dyed fibers. The key principle is to determine the spectral bands that are characteristically distinctive between the dye classes. At first, we show that natural or synthetic dyes can be classified by the reflectance spectra of the dyed fibers. Next, we show that this can be done through a small number of well-chosen spectral bands. We apply sparse logistic regression technique to recognize the discriminating spectral bands, and later a simple support vector machine algorithm (SVM) method is used to classify the dyes of any arbitrary sample considering the recognized bands. The objectives of this paper therefore are of twofold:

1. Determine the wavelength bands useful to classify natural and synthetic dyes.
2. Recommend three optimal bands to achieve a reasonable accuracy.

## 2. Materials and Methods

### 2.1. Samples of Dyed Fibers

**Figure 1:**
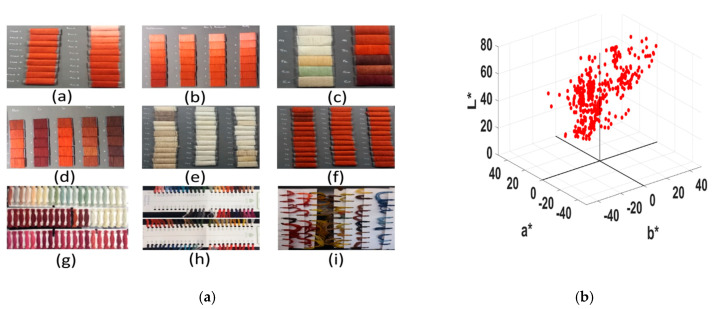
(**a**) Images of all samples used in this study; (**b**) colors of the samples in CIELAB space.

In this study, the reference dyed fiber set consisted of dyed wools as they are frequently encountered in historical textiles [36] due to its ecological nature and human friendly. The sample set included a large number of woolen yarns that were dyed by natural Madder in varying aspects. Please note that Madder is a frequently found and historically important natural dye. It produces color shades varying from pink to black, purple and red if fibers are treated with different mordants salts. The entire sample set contained total 459 woolen threads shown in Figure 1a, and their colors are shown in the CIELAB color space in Figure 1b. We separated the samples into two sets depending on the origins and preparations that are described below. Note that we have used the dyed fiber of red hues of Set-1 to demonstrate the detailed analysis all through this paper.

Set-1: The samples were prepared by dyeing experts in Iran and consists of 216 patches among which 60 patches (Figure 1a(g)) were dyed with acid dyes manufactured by former Ciba-Geigy Ltd., Basel, Switzerland. Other patches were dyed with Madders treated with different mordant salts (Al, Sn, Fe, Cu and Cr) and some patches were dyed without any mordant treatments. The Madder roots were of different ages (average three years), and places (Nain, Khur-Biabanak and Bafq in Iran, and Mediterranean area). The roots were finely powdered in local traditional gristmill, and different recipes were followed to dye the woolen yarns [37]. At the time of this study the average age of the samples was around five years. Figure 1a(a) shows patches with variable concentrations of Madders (5%, 10%, 20%,...,100%). Figure 1a(b) shows four columns of samples where each column was characterized by Madders originated in four different places. Figure 1a(c) shows patches before dyeing and after dyeing treated with mordant salts. The samples in Figure 1a(d) were characterized with treatment of mordants salts and dyed with variable concentrations of Madders. Figure 1a(e) shows bare wools of different tones and thickness, and Figure 1a(f) shows them after dyeing with the Madder.

Set-2: These samples were collected from a house of Arts and Creative Handicrafts (Wetterhoff, Finland). At the time of this study, the age of the sample set was over 11 years. Among 243 samples, 90 samples were colored by synthetic dyes (Figure 1a(h)) and the rest of them (Figure 1a(i)) were colored by the dyes of natural origins (Madder, Indigo, Weld, Oak barks etc.). Different recipes and mordant treatments were applied for dyeing the woolen yarns.

### 2.2. Measurements

#### 2.2.1. Spectral Imaging

**Figure 2:**
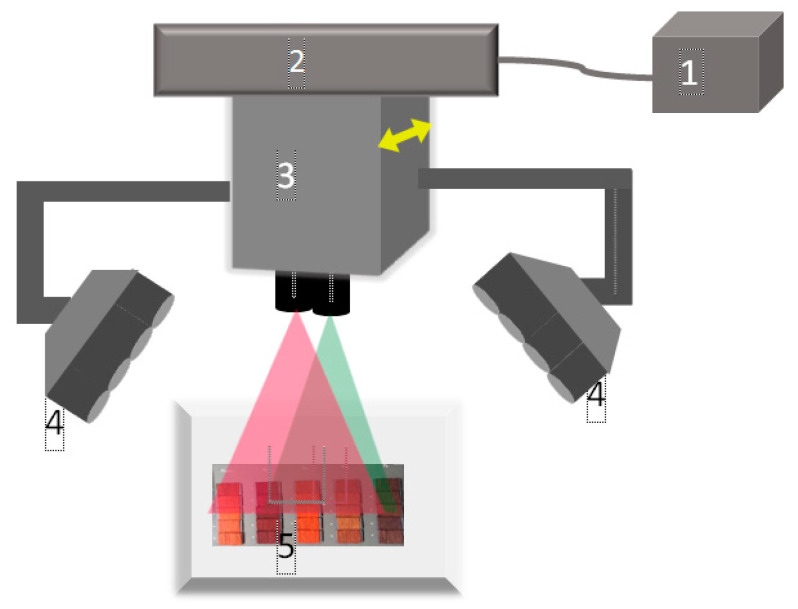
Schematic diagram of the spectral imaging system: (1) the control unit, (2) the linear conveyor, (3) the container of spectral cameras, (4) the halogen lamps and (5) the samples.

The images of the samples were captured with a spectral line scan system (Specim, Oulu, Finland) that had two cameras covering the V/NIR and SWIR spectral range, independently. Two sets of halogen lamps (35 W each, 45/0 degree geometry) mounted in a separate casing moved with the camera to illuminate the samples (Figure 2). The scanning speed was fixed at 25 mm/s for both cameras. The internal shutter of the camera was closed to acquire a dark image, and a Spectralon (Specim Ltd., Oulu, Finland) white plate was measured for the reference image. Table 1 describes other specifications of the system.

#### 2.2.2. Image Pre-Processing

The imaging procedure produced three-dimensional (3D) cube for each measured sample. Each 3D cube contains the spatial information in 2D and spectral information in the third dimension. The effect of dark current and illumination variations across the line scan was removed through the computation of spectral reflectance image H as follows.

(1) $$H=\frac{Sample\;Image-Dark\;Image}{White\;Image-Dark\;Image}\cdot W$$

where the matrix W contains known reflectance factors of the reference white. The average wavelength sampling interval was 3 nm and 6 nm for V/NIR and SWIR range but the interval was not equal for all wavelength regions. Moreover, very large data size of RAW format images made the processing unfeasible. Therefore, a spline interpolation method was applied to resample the reflectance in the spectral dimension at 10-nm intervals. The final spectral range was 400–1000 nm for V/NIR and 1000–2500 nm for the SWIR wavelengths at the step of 10 nm.

### 2.3. Data Analysis

#### 2.3.1. Reflectance Spectra

**Figure 3:**
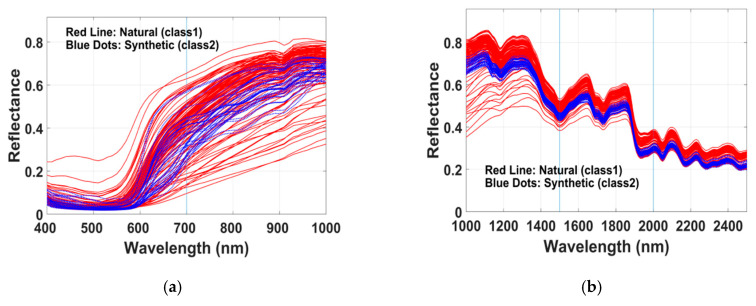
Reflectance spectra of 114 yarns dyed with natural Madder (solid red lines) and 34 samples dyed with synthetic red dyes (dotted blue lines): (**a**) the V/NIR range, (**b**) the SWIR range.

The spectral image of each palette was visualized in the computer display as the RGB image rendered by the spectral envelope visualization method [38]. Then we manually selected multiple pixels at random spatial locations of each patch and stored the mean reflectance spectrum of that patch. To reduce the effect of unevenness of the surface, we collected the pixels from the ridges of the yarns. This process generated reflectance spectra as vectors with a dimension of 61 for the V/NIR image and 151 for the SWIR image. The known class information of each sample was associated with the corresponding reflectance vector. Figure 3 shows the reflectance spectra of red colored samples of Set-1. The solid lines in red color in the plot represent the natural class (Madder) and the dotted lines in blue color represent the synthetic class. Notice that in contrast to the spectra in SWIR range (Figure 3b) the spectra of both the classes overlap inseparably in V/NIR range (Figure 3a).

#### 2.3.2. Spectral Band Selection Procedure

To identify the wavelengths with the most discriminatory power, we partitioned the range of wavelengths into separate regions. The regions in the V/NIR range were: (400–700 nm), (700–1000 nm) and (400–1000 nm), and the regions in SWIR range are: (1000–1500 nm), (1500–2000 nm), (2000–2500 nm), (1000–2000 nm), (1500–2500 nm) and (1000–2500) nm. This partition of wavelengths into groups was performed intuitively visualizing the spans of reflectance in wavelength scale (Figure 3). We used the reflectance spectra of 70% randomly chosen samples of each class to serve as the training data for the spectral band selection process. A sparse logistic regression technique with a Bayesian regularization approach was applied to select the useful bands for discriminating the dye class [29,30]. A particular band was selected if the corresponding logistic regression coefficient was a positive value. The details of the model and the optimization process of the regularization parameters are presented in [39,40,41,42]. This approach has been used for feature selection in high-dimensional data classification in various application areas. In this study, wavelength bands were selected using a one-versus-all implementation of the algorithm [43].

#### 2.3.3. Classification

Classification of the spectral bands was performed using a binary classification support vector machine (c-SVM) model. A polynomial kernel of order 3 was chosen enabling standard data normalization through a trial and error basis. A leave-one-out cross-validation method [39] was applied on each subset of spectral bands for evaluating the classification accuracy. Table 2 lists the settled parameters for this study. Each subset of bands in the V/NIR or SWIR range was considered independently to determine the class (natural or synthetic) and to evaluate the accuracy.

#### 2.3.4. Calculation of Indexed Ratio Features

Two ratio indexes calculated using reflectance of three optimal bands can be used as features to achieve high classification accuracy [41]. The bands are determined as a result of searching optimum classification accuracy using all possible band combinations. If the optimal spectral bands are Band1, Band2 and Band3, the formulas are described in Equations (2) and (3). Thereafter, *index1* and *index2* are used as features in the classifier.

(2) $$index1=\frac{r\left(Band1\right)-r\left(Band2\right)}{r\left(Band1\right)+r\left(Band2\right)}$$

(3) $$index2=\frac{r\left(Band1\right)-r\left(Band3\right)}{r\left(Band1\right)+r\left(Band3\right)}$$

## 3. Results

This section has three parts. In the first, we present the results taking into account the spectra (that represented in Figure 3) of fibers dyed with only natural Madder and synthetic red dyes. The purpose is to evaluate the aptness of the method and interpret the results. In addition to that, since natural Madder is a frequently and historically used colorant in cultural objects, we want to separately report the classification results. In the second, we present the results of all the samples colored with various natural and synthetic dyes. Thereafter, we represent the results of optimal (three) band selection process and their accuracy.

**Figure 4:**
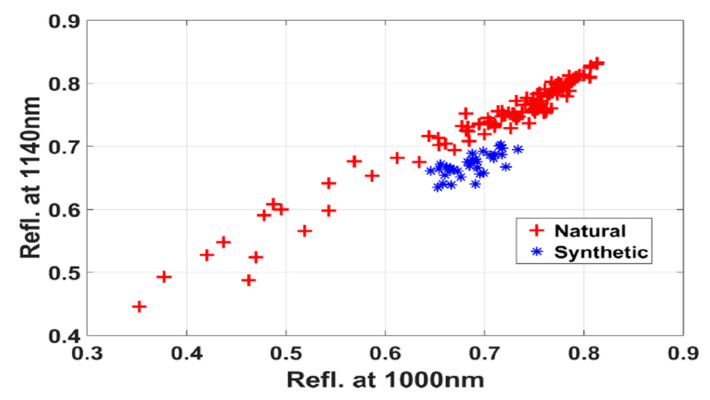
Scatterings of the samples (cases- 4,7,9) in the reflectance space of 1000 nm and 1140 nm to give a visual support of obtained 100% classification accuracy.

The results in Table 3 were yielded by the spectra of the fibers dyed with the natural Madder or synthetic red dyes. The table shows identified discriminatory wavelength (WL) bands that are useful for recognizing the corresponding class and the leave-one-out classification accuracy. The selected bands in each group in the third column are shown according to the order of selections by the sparse logistic regression algorithm. Note that the same bands have been selected in different group while the spectral range was expanded to new regions implying their importance in discriminating the class. However, for case-2 and case-6 only one band was selected. In those cases, either the classification algorithm did not converge or a whole class of samples was misclassified. These cases are marked by dashes. Here, natural class error 6.1% means that 6.1% samples dyed with synthetic dyes have been misclassified as natural class samples. Except case-6 note that in other cases of SWIR range, the classification accuracy is over 99% with the kappa value around 1.0. The results thus evidently show that the bands in the SWIR range have a better discriminating power than the V/NIR range. In cases-4,7,9, the obtained classification accuracy is 100% for the selected bands at 1140 nm and 1000 nm. To check the agreement between the dye classes through a visual support, in Figure 4 we show the scatterings of the samples in the reflectance space. This figure demonstrates that a distinct class separating boundary line can clearly separate the members of both classes.

As the initial results and observation were convincing, the same technique was applied to the spectra of all the samples comprising Set-1 and Set-2 altogether. Table 4 represents the results. The obtained best accuracy is 97.4% (kappa 0.94) in case-9 with less than 3% miss classification error in either of the classes. In case-9, nine bands were selected by the algorithm. But notice that with six and five bands case-7 and case-8 achieved accuracy just less than 1% compared to the accuracy of case-9. The classification accuracy depends on the number and order of the selected bands.

**Figure 5:**
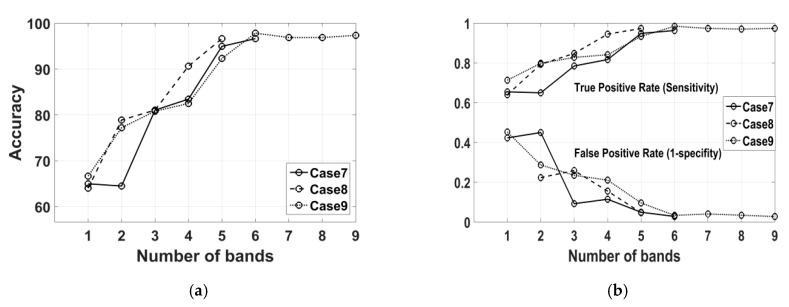
Performance measures in increasing order of adding the selected bands for cases 7–9: (**a**) classification accuracies, (**b**) the TPR and FPR rate.

For the best three groups of spectral bands (cases 7–9), Figure 5a shows the classification accuracies and Figure 5b shows the sensitivity or true positive rate (TPR) and the false positive rate (FPR), in increasing order of adding the number of bands. For the first six bands in case-9, the maximum accuracy (97.8%) and maximum TPR were obtained, but adding last the three bands, in fact, slightly degraded the performance (97.4%). For case-7, adding the second band did not change the accuracy but adding the third band dramatically increased the accuracy and decreased the FPR. Due to this observation we linearly searched to determine the top bands from the selected bands in cases-7,8,9 yielding a high accuracy.

Three bands at 1640 nm, 2330 nm and 1480 nm were identified that produced the maximum 90.1% accuracy. We calculated the two ratio indexes using these optimal bands (Equations (2) and (3)) and used as features in the classifier. Randomly chosen 70% samples were used to train an SVM classifier with a simple quadratic kernel. The remaining samples were used to validate the classification accuracy. The average result of 100 iterations is shown in Table 5.

## 4. Discussion

**Figure 6:**
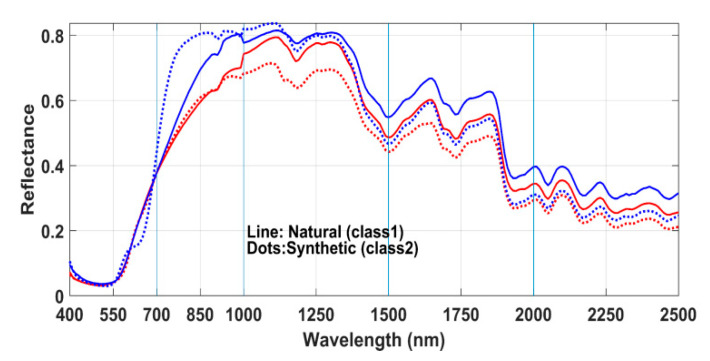
Average reflectance spectra of several patches with Red hue of natural and synthetic class both from set-1 and Set-2.

In this study, we show that machine learning based computational methods can identify the relevant spectral bands to discriminate the dye classes. For this experiment, we studied a large number and wide variety of woolen fibers dyed with various natural and synthetic dyes. The results demonstrate that, in general, the spectral bands in SWIR range (1000–2500 nm) have higher class discriminating power compared to the bands in V/NIR range (400–1000 nm). In any performance aspect, the selected bands in SWIR range yields superior result (Table 4). It is also noteworthy that three optimal bands in SWIR range can achieve around 90% classification accuracy. It was not straightforward to extract the class discriminating bands as the measured reflectance spectra are not molecular specific. Rather the reflectance spectra of dyed fibers encode convoluted information of mixed molecules [29]. Figure 6 demonstrates that reflectance of dyed wool fibers are overlapping in visible region [44], on the other hand, reflectance are non-overlapping in some wavelength regions in NIR to SWIR range. The proposed computational technique in this study was successful to automatically recognize the effective class discriminating bands in SWIR spectral range.

The spectra (Figure 6) and the obtained results demonstrate that the natural and synthetic dyes have distinct characteristics difference in optical absorption in SWIR region. This statement is supported by some existing literatures. Interestingly, it has been reported in [14,22] that chromophores of many synthetic dyes (e.g., azo dyes) are absorbing in infrared range. In [36], it has been revealed that some natural dyes can change the photoluminescence property of wools in visible range, which are not present in undyed wool. Moreover, synthetic dyes change subtle chemical structure (e.g., in keratin) of wool fiber [14]. There are many dye chromophores which absorb in the infrared or near-infrared region [5,22,24]. Therefore, in this context of our paper, it may be interesting to look at the selected bands and their behaviors. Several comments about the bands selected for the classification (Table 3) are worthy to mention.

**Figure 7:**
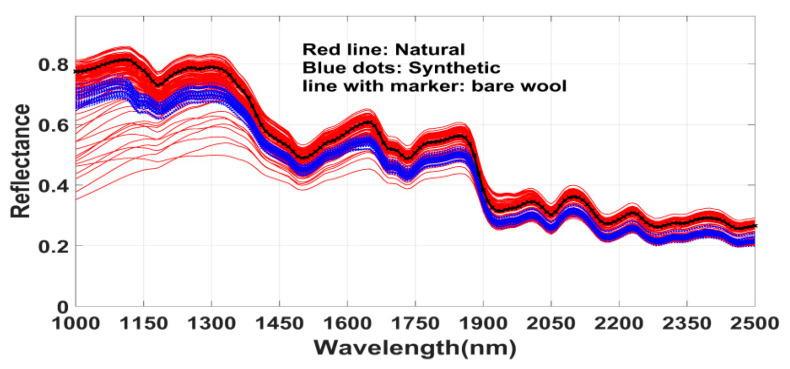
Reflectance spectra of bare wool and fibers dyed with natural Madder and synthetic red dyes.

As a case study, the subset of samples of Set1, attributed to natural Madder and synthetic red dyes, were examined. Figure 7 shows the spectra (in SWIR range) of the undyed wool fiber (black line) and the dyed fibers of both classes. Visually the shape of spectra of dyed fibers looks almost similar to the spectrum pattern of undyed wool. It also illustrates that in contrast to a large number of naturally dyed fibers, fibers with synthetic dyes have less reflection values.

**Figure 8:**
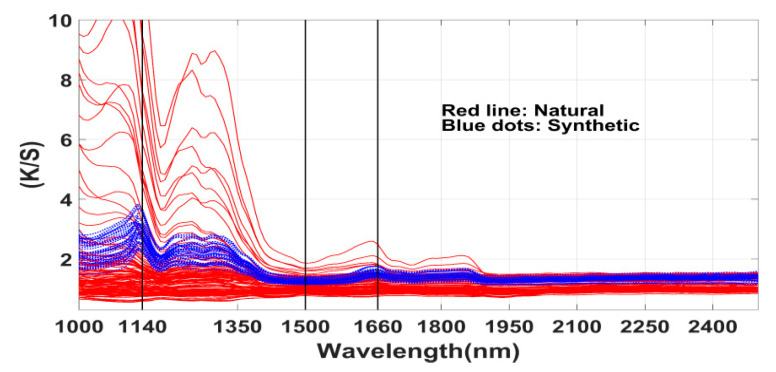
Reflectance spectra transformed to ratio of absorption and scattering coefficients (K/S) and the locations of selected bands (1140 nm, 1500 nm, 1660 nm) indicated by vertical lines.

For a better visualization the reflectance was converted to the ratio of absorption and scattering coefficients (K/S) by the standard Kubelka–Munk formula [45] and illustrated in Figure 8. Note that the selected bands at 1000 nm, 1140 nm, 1500 nm, 1660 nm for this subset of samples produced almost 100% classification accuracy (Table 3). This figure illustrates that the logistic regression algorithm reasonably identified the discriminating spectral bands. For example, in contrast to the natural class the spectra of synthetic class show consistent differences between bands at 1140 nm. It is known that natural dyes weakly absorb in the infrared region. But some spectra show high absorption between 1000–1350 nm possibly due to the effect of mordant salts and/or interesting fluorescence properties. The spectra of both classes are simply flat for the bands in case-7 (2000–2500 nm). In this range, the algorithm selected just one band indicating lack of a pair of discriminating bands.

**Figure 9:**
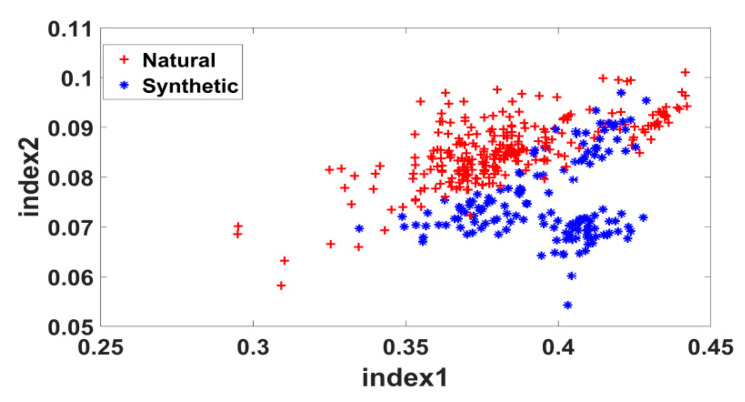
Scatterings of all samples from both classes in the space of normalized indexes.

In the Results section we have reported the data and illustrations in details so that the validation of the proposed technique can be envisaged. It was found that top three spectral bands in (1000–2500nm) wavelength range satisfactorily classify the natural or synthetic dye class applied on woolen fibers. The general technique was to use a priori class information in determining the most discriminative bands and then apply a classifier on those bands for new samples. The optimum spectral bands were 1640 nm, 2330 nm, and 1480 nm. Figure 9 represents the ratio indexes of the samples calculated using those optimum bands (Equations (2) and (3)) and validates the obtained accuracy (90.1%) reported in Table 4. The samples are separated in a useful pattern in the calculated feature space, and a SVM kernel draws a decision boundary that produces almost 90% accuracy.

## 5. Limitations and Future Directions

This paper proposes a novel approach for non-destructive application of spectral imaging to investigate historical textiles in-situ through an empirical study of mockup samples. It should be noted that this work is the very first attempt to classify the textile dyes using spectral camera to the best of authors’ knowledge. During this study the authors did not find any such literature to make a comparative study of the obtained results. Besides the technology the same samples/datasets are to be used for an acceptable comparison, but any such public dataset was also not available at the time of this study. The closest literature was [29,31,33] where the authors applied spectral imaging to identify and mapping of pigments materials, in reading palimpsests and in visualization of coatings in paintings artwork.

Although the number of samples used in this experiment is enough for a primary study for computational method development [27], in the future, investigations will be performed with an increased number of samples in each class with different types of dyes applied on them following different preparations, mordant treatments and dying techniques. The results reported in this paper were obtained examining only wool fibers that were prepared by the experts in dying process and heritage science. The used dataset can be supplied upon request by other researchers.

It is observed that the absorption coefficients of the materials in dyed fibers are wavelength dependent. So, tuning the imaging bands from V/NIR to SWIR, selective imaging of different layers can be performed to visualize and investigate target areas regarding any subtle question. Inexpensive handheld device can be built with selected top three discriminating bands to real-time and non-destructive mapping of the dye class in a large area of a cultural object.

Other works in the future will be to apply the same technique considering various types of fabrics (e.g., cotton, linen, silk) with varying ages. Also, it will be worth to investigate the reflectance of samples affected by oily, protein stains. The observations and result in this study encourage further investigating the data in order to detect and identify mordant salts. Moreover, different subclass, origins and age of the dyes can be studied that have profound applications in history and authentication of the textile objects and is worthy of scientific investigation.

## 6. Conclusions

In this paper, different natural and synthetic dyes on wool fiber were studied. It is shown that fiber dyes can be classified as natural or synthetic with high accuracy using spectral imaging in the SWIR (1000–2500 nm) range. A simple machine learning approach was applied to recognize and use the most informative bands in classifying the fiber dyes. The spectral bands in V/NIR (400–1000 nm) range appeared not as effective as SWIR range. The results show that nine bands in SWIR range can achieve 97.4% classification accuracy and kappa 0.94. Interestingly, top three selected bands can produce around 90% classification accuracy and kappa 0.78. Within subgroup of samples, i.e., wool fiber dyed by natural madder or synthetic red pigment, the separation between natural and synthetic dye is possible with the pair of wavelengths 1000 nm and 1140 nm. In this range the reflectance from wool fiber should not be disturbing. In the SWIR range the signal detected comes from deeper and more complex scattering profile compared to VNIR. Although, the wavelengths used in the classification are not disturbed by the characteristic peaks of wool fiber, further studies are required to fully explain classification between natural and synthetic pigments. This study thus opens the opportunity to further work on spectral imaging in non-destructive study of colored textile fibers in exploring more questions about the fibers and the dyes that they contain.

## Figures and Tables

**Table 1:** Measurement specifications of the spectral camera system.

	V/NIR Range	SWIR Range
**Camera**	Zyla 5.5 sCMOS; Andor Tech., UK	LVDS-100; Specim, Spectral Imaging Ltd.
**Lens**	V18.5 – f/2.4 091101 Specim Ltd.	OLES15-f1/2.0 052301 Specim Ltd.
**Spectrograph**	ImSpector V10E; Specim, Spectral Imaging Ltd.	ImSpector N25E; Specim Spectral Imaging Ltd.
**Data Form**	Spectral radiance, 386–998 nm at 3 nm intervals	Spectral radiance, 933-2538 nm at 6 nm intervals
**Standard White**	Spectralon^®^ reference plate (Specim Ltd.)	Spectralon^®^ reference plate (Specim Ltd.)
**Light Source**	2 set halogen lamps (35 W each), 45/0° geometry	2 set halogen lamps (35 W each), 45/0° geometry
**Spatial Resolution**	6.5 micrometer	30 micrometer
**Sample distance**	395 mm	380 mm
**Exposure time**	9.1 ms	1.6 ms

**Table 2:** The MATLAB configuration parameters for fitcsvm function.

Parameter	Value
Kernel Function	polynomial
PolynomialOrder	3
KernelScale	1(default)
Standardize	True
Leaveout	On
Solver	Sequential Minimal Optimization (SMO)

**Table 3:** Leave-one-out classification performance considering the spectra of fibers dyed with natural Madder or synthetic red dyes.

Case	WL Range (nm)	Selected Bands (nm)	Classification Accuracy (%)	Cohen’s Kappa	Natural Class Error (%)	Synthetic Class Error (%)
1	400–700	580, 590, 400	90.5	0.73	6.1	20.6
2	700–1000	700				
3	400–1000	580, 400, 590	90.5	0.73	6.1	20.6
4	1000–1500	1000, 1140	100	1.0	0	0
5	1500–2000	1500, 1660	99.3	0.98	0	2.9
6	2000–2500	2000				
7	1000–2000	1000, 1140	100	1.0	0	0
8	1500–2500	1500, 1660	99.3	0.98	0	2.9
9	1000–2500	1000, 1140	100	1.0	0	0

**Table 4:** Leave-one-out classification performance between natural and synthetic dye class considering spectra of all the samples.

Case	Selected Bands (nm)	Accuracy (%)	Cohen’s Kappa	Natural Class Error (%)	Synthetic Class Error (%)
1	640, 700, 570, 480, 400	91.6	0.82	7.5	10.0
2	810, 780, 700	79.9	0.52	26.6	8.7
3	640, 700, 810, 800, 560, 450, 1000, 710, 600, 400	94.0	0.87	6.4	5.3
4	1460, 1470, 1000, 1140	93.3	0.86	5.6	8.0
5	1640, 1690, 1500	87.3	0.70	18.7	2.0
6	2000, 2500, 2290	89.9	0.78	7.1	15.3
7	1470, 1460, 1730, 1000, 1140, 1980	96.9	0.93	3.8	2.7
8	1640, 2300, 2500, 1920, 1500	96.6	0.93	2.6	4.6
9	1140, 1920, 1480, 1910, 1000, 2500, 2330, 1130, 1450	97.4	0.94	2.6	2.7

**Table 5:** Average results of 100 iterations using normalized ratio indexes as the features.

Case	Top Three Bands (nm)	Accuracy (%)	Cohen’s Kappa	Natural Class Error (%)	Synthetic Class Error (%)
2 ratio indexes	1640, 2330, 1480	90.1	0.78	9.8	10.0
